# The experiences of dietitian’s working in care homes in England: a qualitative study

**DOI:** 10.1093/ageing/afac006

**Published:** 2022-02-13

**Authors:** Vittoria Romano, Catherine J Minns Lowe

**Affiliations:** Nutrition and Dietetics Department, Central London Community Healthcare NHS Trust, Lisson Grove Health Centre, Gateforth Street, NW8 8EG; Department of Allied Health Professions, Midwifery and Social Work, School of Health and Social Work, University of Hertfordshire, Hatfield, UK

**Keywords:** dietetics, nutrition support, health care delivery, qualitative, older people

## Abstract

**Background:**

The provision of appropriate nutritional care in care homes is a priority for health services in England. There is limited evidence demonstrating the role of dietitians within older people care homes. This study explores the experiences of dietitians working with care homes for older people in England.

**Methods:**

A qualitative study using semi-structured face-to-face or telephone interviews was conducted. Criterion and snowball purposive sampling recruited six dietitian participants. Interviews were audio recorded and transcribed verbatim. A reflexive diary was completed, and data analyses followed interpretative phenomenological analyses. Constant comparison, code–recode audits, independent coding by a supervisor, supervisory support and peer review were used to promote rigour.

**Results:**

Two key themes and three subthemes were identified: Theme 1 is collaboration with multidisciplinary team (MDT) professionals and its two subthemes are as follows: using support strategies (pathway/standards implementation, training/education and resident dietetic assessment) and delivering value (by benefitting more residents, demonstrating unique dietetic skills, nutritional prescription savings and meeting other professional’s knowledge gap). Theme 2 is communication with MDT professionals and its subtheme is the understanding of the dietitian’s role and of nutritional care.

**Conclusion:**

Dietitians believe that they play a key role in supporting care homes with nutritional care, identifying themselves as experts and leaders, working with MDT professionals. The findings highlight the importance of a consistent approach to managing nutrition and the need for dietitians to share outcome data to improve the limited evidence-base. There is a need to agree a defined dietetic service provision to care homes in England.

## Key Points

- Dietitians working with care homes can have significant and varied input into care homes, but there is a need for a nationally defined role.
- The dietitian’s role in care homes is under-researched and, in this study, interpretive phenomenological analysis has enabled dietitians’ experiences to be explored.
- Collaboration with multidisciplinary team (MDT) professionals is a key part of the dietitian’s role and involves using a diverse range of support strategies and opportunities to add value to the care of residents.
- Communication with MDT professionals is important to ensure the dietitian’s role is understood as well as the nutritional needs of residents.
- Investment in dietetic services could support the implementation and delivery of the enhanced health in care homes framework in England.

## Introduction

The estimated cost for disease-related malnutrition in England is £19.6 billion to health care annually, 52% of which is for older people [1]. Care home residents represent a disproportionately costly sector of the population [1]. Older people in care homes are also 50% more likely to be admitted to hospital than their community-dwelling counterparts [2, 3]. Improving the management of malnutrition could support the financial efficiency targets of the National Health Service (NHS), with the potential to provide the third biggest saving in health care costs [1, 4, 5]. In care homes, it is likely that efforts to target and manage malnutrition will achieve significant cost savings by reducing admissions and length of hospital stay, while improving the quality of life and autonomy of residents [6].

The NHS Sustainability and Transformation Plans (STP) for care homes have recognised the importance of nutritional care from primary care teams, including a dietitian as a key member [7]. However, a recent systematic review revealed little research defining the dietitian’s role in care homes in England with no legal requirement for care homes to involve a dietitian [8]. This is in contrast to North America where dietitian involvement is mandatory [9, 10]. In England, STP implementation is variable [11], and where dietetic provision was surveyed in one English locality, only 50 out of 115 care homes used dietitian services [12].

Dietitians assess and treat nutritional issues, by educating and giving personalised advice to the people who use their services, their carers, health and social care professionals and the voluntary sector [13]. The Care Quality Commission (CQC) [14] reported a correlation between care homes that sought dietitian services, specifically for menu planning and advising on at-risk residents, and meeting nutritional care standards. This indicates that dietitians could contribute to good nutritional care in care homes, within the context of the care home framework and its subsidiary aims of reducing duplication and costs [7].

It is not known if the input of dietitians in the English care home setting improves nutritional care. A first step of enquiry might be to explore the current roles and experiences of dietitians to discover the extent they work with care homes to promote professional discussion and learning and help identify areas for development. This study aims to explore the experiences of dietitians working with older people care homes in England, including how they view their role, how they think other professionals view their role and how they view the future role of dietitians working with older people care homes.

## Methods

### Design

A qualitative study using the interpretive framework social constructivism with an interpretative phenomenological analysis (IPA) approach was conducted [15]. This approach to analyses seeks to understand the meaning people give to their experiences of a particular phenomenon and is useful when little is known on a topic [16]. As the dietitian’s role in care homes is an under-researched area, an exploratory qualitative study using IPA was appropriate. The interviewer’s (VR) background of working as a dietitian in care homes facilitated professional discussions and interpretation of people’s experiences [15]; care was taken to use open, non-leading questions with participants, and a reflexive diary was used to try and identify the interviewer’s assumptions and beliefs about the topic before and during the study. Analyses can produce rich, detailed understanding of participant’s shared experiences that individuals can relate to their own practice [17]; they do not produce transferable findings to clinical practice [18].

Ethical approval was issued by the University of Hertfordshire, Health Science Engineering & Technology ECDA Ethics Committee (aHSK/PGT/UH/03341(1)).

### Sampling and recruitment

Purposive criterion sampling was used to ensure that participants had experienced the phenomenon being explored [19]. The British Dietetic Association’s (BDA) Specialist Groups with known interest in older people’s nutrition [20, 21] emailed invitations to members. Members were encouraged to share the invitation with others meeting the inclusion criteria as a form of snowball sampling, to encourage the recruitment of non-group members [22]. Information sheets were emailed to those who had expressed interest in participating, and opportunity to ask questions was provided. Interviews were arranged with willing participants and written consent collected prior to the interview (using pre-paid return envelopes). Participants scheduled their interview and could choose between face-to-face, telephone or video calling to avoid geographical recruitment barriers and encourage rapport and participant comfort to explore experiences [23]. Participants were asked for their length of service as a dietitian for context. Information regarding job title was obtained; however, due to some individuals potentially being identifiable, these have not been disclosed. Anyone who directly worked with the researcher was excluded as shared experiences could risk study credibility [24]. It was estimated that six participants might provide the rich, in-depth understanding of the participants’ experiences, and this was set as a provisional target [15, 25]. Interviews were digitally audio recorded.

### Data collection

A semi-structured interview guide (Appendix 1) was developed and refined (V.R.) with assistance by her MSc supervisor (C.M.L.). The guide included descriptive questions to support rapport building, open questions to obtain views, and evaluative questions and prompts to support data interpretation [15]. The schedule was used flexibly to facilitate a participant-led discussion, and prompts were used to encourage the in-depth information needed for interpretation of experiences [16]. The first interview was used as a pilot and discussed in depth with C.M.L. to make sure rich data were obtained. Six interviews were held between July and November 2018 (V.R.). A reflexive diary was recorded throughout (V.R.) to enhance credibility by recognising and minimising researcher influence in interpretations [26].

### Data analyses

Data were fully transcribed (V.R.). Audio recordings were listened to and transcripts read until familiar [15]. Line-by-line notes were added to the transcripts’ right-hand margin, which described the experiences reported and the researcher’s interpretation of their meaning. These notes were then analysed in sections to identify the patterns of meaning that were annotated in the left-hand margin as codes. A reflexive diary was used to bracket each experience and minimise influence from other participants to improve credibility [17]. The first interview transcription and coding was peer reviewed by C.M.L. and discussed.

Early codes were collapsed into themes by identifying patterns of meaning discussed in depth (V.R., C.M.L.). A code–recode audit of each dataset was completed to minimise any previous influence that may have compromised the idiographic focus and to improve the confirmability of the findings [27]. Patterns of similar meanings across all datasets were identified and developed into themes. Data collection and analysis was completed with six participants as this number was realistic for the MSc time frame and facilitates rich data collection rather than risking superficial analysis with larger samples [18]. Data saturation was not achieved due to the time frame and is not usually required in IPA as the focus is on the individual’s account [28].

Analysis was iterative and maintained IPA’s double hermeneutics, by using constant comparison of data, codes and themes, involving researcher interpretation of the participant’s experience [16]. Discussion of themes involved literature searching to encourage further interpretation of data in relation to current theory. Strategies to encourage rigour included peer support for interview techniques and peer reviewing the coding processes (C.M.L.) [27]. After completion of the MSc, the supervisor coded all transcripts and cross referenced these against the initial codes, categories and emerging themes developed by V.R. to promote trustworthiness of data interpretation [29, 30].

## Results

The characteristics of six participants are presented (Appendix 2). Two participants were interviewed face-to-face: one at home and one at work (management approval gained). Four participants requested telephone interviews. The interview duration ranged between 33 and 49 minutes (average 43 minutes). To encourage data completeness, participants confirmed the end of the interview [15]. There were no withdrawals. The number of early codes identified from the individual data sets ranged between 16 and 25, the mid-codes 4–7 and final codes 2–3: individual data are presented (Appendix 1).

**Diagram 1:**
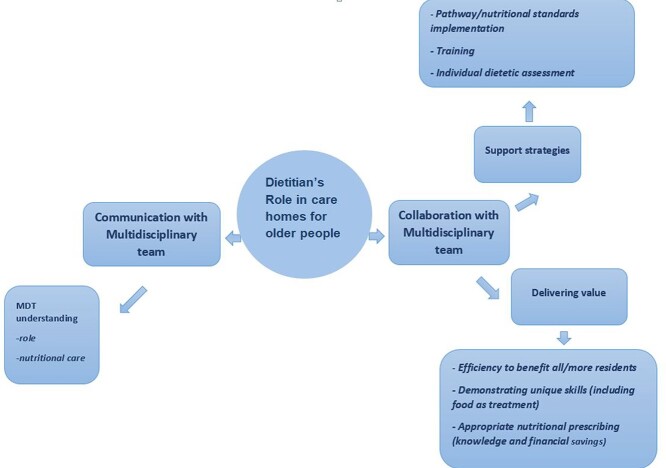
Summary of two main themes and subthemes.

Patterns of similar meaning were identified across the final codes from all data sets resulting in three key themes. Two themes directly met the study aim and are presented here (Diagram 1): communication with multidisciplinary team (MDT) and collaboration with MDT professionals. The themes are presented in the narrative alongside the subthemes: support strategies, delivering value and MDT understanding and quotations from the participants. The third theme explored process issues and is not presented here.

## Communication with MDT

### MDT understanding

#### Role

MDT understanding of the dietitians role needed to improve as they: ‘didn’t realise that that’s how complex it all is’ (I/V1) and so learnt that they needed to ‘tell them what my role is’ and be ‘really proactive…bang on every door…become known and nutrition is on agenda’ (I/V1). Another participant explained working with the MDT improves role value ‘[MDT] had a lot more input from dietetics…they value us but only when they get the chance to know us’ (I/V3). Communication with the MDT provided supportive reassurance to care home staff ‘the reassurance that they are doing the right thing’ (I/V2) and helped to promote nutritional ‘messages [which] are consistent’ (I/V2) across the team.

Several dietitians who had a prescribing support role perceived the MDT viewed their role negatively but that ‘it gets better with the conversation’ (I/V4) and other times poor understanding negatively impacted the role:

some of them…are really positive and others you can sense the negativity towards the role (I/V4)

*…*my acute colleagues find the role more difficult to understand…advising quite large prescriptions…we had to pick those things up…it’s fallen a little bit on deaf ears (I/V4)*.*

acute dietitians don’t really understand…nobody thinks…there are more cost-effective options available (I/V1).

#### Nutritional care

One participant found that the MDT (including care home staff) had incorrect beliefs on appropriate nutritional care in older people and they saw their role as:

…trying to help people to understand …what we can and should be doing…for the resident’s quality of life (I/V2).

Explaining nutritional care to the MDT was helpful:

usually as long as I explain why…I’m going down that route…they’re usually fine with it then (I/V5)

Some participants’ though reported difficult conversations with the MDT around their role focus:

having a financial target is not the best way to improve the quality…that’s really hard to get across (I/V1)

Participants also recognised that the workload of care home staff is ‘incredibly demanding’ and that providing additional nutritional care could place ‘extra pressure’ (I/V5) and responsibility upon staff. Listening, respect and communicating as a team though could lead to ‘working together’ (I/V3) meaning ‘we can do the very best for residents’ (I/V2).

Additionally, proving the effectiveness of preventative, non-patient facing work is difficult:

dietitians struggle with it because you’re not seeing patients…so what are you actually doing…what’s it really achieving (I/V6).

It could be a ‘huge learning curve’ (I/V 1) for the dietitian to work in the complex care home setting.

## Collaboration with MDT

Developing collaborations takes time and can be ‘very slow’ (I/V1) and ‘very…variable’ (I/V3) amongst different care homes. It was recognised that change ‘perhaps adds an element of stress’ (I/V 5) and participants described how collaborations were developed.

### Support strategies

#### Pathway/nutritional standards implementation

Most participants collaborated with care homes staff to support them with delivering quality nutritional care to residents. Some worked with care homes to improve care quality through implementation of a food-based pathway and to reduce nutritional prescribing:

…reviewing individual patients…inefficient…better to focus on…quality of their nutrition provision…then…little need for prescribing nutritional supplements (I/V1).

Some participants used nutritional standards developed in collaboration and endorsed by the wider MDT who were perceived to have the power and potential to influence uptake of nutritional standards by care homes ‘council…withhold money, if they feel the care homes are failing in that area’ (I/V4).

One dietitian used audit to measure standard compliance which is linked to funding:

every year once we’ve trained up a home we audit them…18 quality standards…if they fail…relate to £70,000 of funding (I/V6).

Audit was not discussed by the other participants, although one was CCG based which they found helped their role as it’s a position of influence with access to other influential stakeholders:

…being part of the CCG really helps…work with our quality team [to]…according to our pathway (I/V1)

#### Training

Care home staff training was used by most participants as a strategy to support pathway/standards implementation:

…homes that had training got phenomenally better outcomes…homes that didn’t … worsened their nutritional care…higher usage of supplements (I/V6).

However, one participant found that training ‘demand. . .exceeds. . .resources’ (I/V4).

Training content was determined from identified improvement areas *‘*a lot of inaccurate heights…that’s another area that we look at…training’ (I/V4). Another trained catering staff as:

…couple of the cooks…brought like women’s weekly…never realised they were doing anything wrong…it’s the cooks we needed to target…[this training] delivers the biggest impact on changes in the homes (I/V6).

Care home staff were described as needing to feel empowered to deliver first-line nutritional care and so training ‘runs as quite interactive’ with ‘additional support to the staff’ (I/V3) and another ‘have the home manager [attend] because the cooks didn’t feel empowered to make changes’ (I/V6).

One dietitian felt their role would ‘be more effective…[with] regular training’ (I/V5) but did not train in their current role. Instead of training, another participant described the education of care home staff and wider MDT as:

One of our most important roles is almost working through other people…if you can meet with lots of different health and care professionals and…spread the message, every person that they see…you can achieve an awful lot more doing it that way (I/V2).

Training of the wider MDT was also beneficial as:

Local Authority inspectors…I…did the training with them…it’s been great because you’ve not only got us saying it, you’ve got all of the inspectors now singing off the same hymn sheet…they are asking them questions and it’s just reinforcing those messages out there (I/V6).

Consistency of nutritional information was again important ‘so that we are all saying exactly the same thing…the right thing’ (I/V1) which led to ‘homes… feeling more confident’ (I/V1) to implement first-line nutritional care.

Some participants supported GPs with appropriate nutritional prescribing by completing ‘…a full assessment on these people on behalf of the GP’ (I/V4). Another worked in a CCG and considered resident reviews as inefficient to meet prescribing savings, they focused on nutritional pathway implementation as this helped the community dietitians who already completing individual resident assessments:

what made a big difference…dietitians…feeling they got the support and back up of the clear pathway…70 were on prescriptions but I think there’s only 30 this year (I/V1).

#### Individual dietetic assessments

Some participants considered individual reviews as inefficient as ‘seeing one individual resident…effectively you are repeating yourself*’* (I/V2) and that more could be achieved supporting care home staff by providing reassurance when transitioning to food-based treatment: ‘[I] emphasise… I’m not just going to… get them off and disappear into the sunset’ (I/V3).

One participant’s role only involved training and audit; however, there was a desire to integrate with the clinical dietetic team who managed individual residents:

I would love… somehow with the elderly team… we could work a lot closer… I think we could get better results as well… more appropriate prescribing (I/V6).

### Delivering value

#### Efficiency to benefit all/more residents

There was agreement that collaboration with the MDT (including care home staff) delivers value by benefitting more residents.

One participant felt that:

…what dietitians can add … working with staff is huge… I would love to see is an end to… typical… dietetic consultation model (I/V2).

Another felt training the MDT delivered preventative care rather than dealing with reactive dietetic referrals:

…provide better training and education to the staff so that patients…are captured early on (I/V5).

Reducing dietetic referrals was important to create efficiency savings as then ‘we can see those that are more complex…quicker’ (I/V6) as before training they experienced ‘crisis referrals…too late down the ladder’ (I/V6). Another agreed that ‘there’s a role for dietitians to be doing the more specialist stuff… [when] first-line… not working’ (I/V3) as ‘[care home staff] are the best people to do it’ (I/V1) and dietitians can see those that ‘truly need to be seen’ (I/V1).

#### Demonstrating unique skills

Training the MDT allowed dietitians to use their unique skills:

…people are living longer, with more complex medical conditions…the dietitian is going to become… invaluable…doctors haven’t got the skills to… manage that alone…that’s where our expertise is going to come in in the future (I/V4).

Another discussed sharing skills with the MDT:

…[we] need to work together because speech and language therapists…often their understanding of nutritional content…is not that great…but that’s where dietitians absolutely come into their own (I/V2).

#### Appropriate nutritional prescribing

Training and pathway implementation was incentivised by allowing care homes ‘to make direct referrals’ (I/V3). Direct referral was a strategy to manage appropriate prescribing and to ‘take the GP out of the loop … bring them in later once their dietetic…assessments been done’ (I/V3).

Other participants felt that their nutritional prescribing support role was valued by the MDT as ‘It’s an area that they’re not too familiar with so they always appreciate… advice from us’ (I/V5) and produced financial savings:

[they had] noted…savings that we produced…they’ve made it [job role] substantive…they’ve actually realised the value of the role (I/V4).

All participants promoted food as first-line nutritional treatment:

Our unique selling point…don’t lose that because otherwise as dietitians we will lose our role within the NHS, certainly in older people’s care. (I/V2)

Another developed a nutritional prescribing policy as they felt their role was to support the MDT:

make sure that they [oral nutritional supplements] are being used appropriately…a standardised approach that’s using food as treatment as our first approach…we should be responsible…with the health economy (I/V6).

Dietetic supplementary prescribing was a value adding future strategy as dietitians would ‘review them very differently, in a better way’ (I/V6):

nobody will be falling through the gap having the appropriate dietetic advice…time saving, cost saving (I/V4**).**

## Discussion

This study revealed two key themes with subthemes relating to the dietitian’s role in care homes. The emphasis of collaborating and communicating with the MDT was shared amongst participants either as their current role or what the role should be based on their experiences.

### Findings in relation to existing studies

Participants collaborated with different members of the MDT and used a range of strategies. US studies revealed similar findings of resident reviews and staff training, although their role emphasis was on foodservice management such as menu planning [10]. US care homes directly employ dietitians, which may explain why they held management responsibilities within the homes to assist them to meet state regulations [31]. On the other hand, the dietitians in this study were not employed by the care homes and viewed their role in the context of MDT integration, nutritional management of residents and medicines optimisation, meeting the NHS strategy for care homes [32].

The CQC [33] acts as a regulator of services including care homes in England and expects them to meet the nutritional needs of residents without the explicit need of involving a dietitian. They also legally require care homes to provide staff training and maintain a competent workforce. The NHS are striving for consistency of care and offering to meet workforce training to enhance the health of residents in care homes and provide equitable healthcare [7]. This reflects the participants’ view that their role included/should include training to staff and the wider MDT. This suggests a culture of shared responsibility for the wellbeing of residents, despite CQC expectations on care home providers and in contrast to care delivery in the USA.

Training focused on improving nutritional care by accurately identifying those at nutritional risk and using food-based treatment as first-line nutrition support. A qualitative study of community nurses (*n* = 20) identified that they valued nutritional training as it improves confidence to implement nutritional screening [34]. The participants in Green *et al*. [34] also applied professional judgement to care delivery which may not be applicable to the care home workforce as they are not always clinically qualified. Additionally, confidence does not guarantee accuracy; nutritional training can be unsuccessful as it needs to change nursing practice [35] which is important now NHS England’s [7, 32] strategy makes nutritional care a priority.

A review of effective health care in English care homes indicated that relational working, so care home staff feel supported, is more likely to lead to successful change than training alone [36]. This may reflect participants using additional support strategies such as pathway implementation and resident assessments when collaborating with the MDT. Goodman *et al*. [36] used a small expert panel of nine participants and whilst the findings cannot be transferable to all care homes/the field of nutrition, they concluded that care home culture, leadership and resource were also determining factors for pathway implementation. This may explain why some participants adapted their input dependent on the care home’s needs and described their role as educators, suggesting a personalised, supportive approach. The educator role was also expressed by Canadian dietitians who used spontaneous opportunities to empower staff with nutritional care [9].

Participants used support strategies to encourage consistency of nutritional information across the MDT. Consistency across health services and care homes was considered integral to the nutritional care of residents with dementia [37]. Dementia affects 70% of older people presenting problems with eating and reflects those requiring nutrition support [38].

Local authority and CQC inspectors were discussed as key MDT members who could support the consistency of nutritional information. As regulators of care homes [39], collaboration may help influence improvements in nutritional care [40].

Collaboration between professionals can be hindered if roles are unknown [41]; there may be barriers to dietitians’ accessing and collaborating with the MDT due to an undefined role and participants agreed that there was a need for dietitians to self-promote for increased recognition. Additionally the CQC [14] referred to dietitians as experts in a nutrition-specific inspection programme in care homes, suggesting they recognise the dietitian’s contribution to nutritional care, irrespective of role definition. Other participants, as per previous research in Canada, held a similar view identifying themselves as expert leaders for nutritional care which supports Canadian research [9].

Some participants promoted food-based treatment to improve nutritional care and achieve cost-effective nutritional prescribing. The published evidence is inconclusive, one systematic review reports oral nutritional supplements (ONS) as cost-effective (although they did not compare ONS to food based options) [42], another review of literature suggests the evidence for food-based interventions in nutrition support is limited [43]. NICE [44] recommends both food and ONS as suitable treatments for malnutrition. The use of inconsistent definitions for appropriate nutritional prescribing is problematic, incorrect volumes, failure to prescribe or lack of clinical indications limit recommendations for practice [45]. In seven of nine studies included in this review, dietitians led the appropriate prescribing interventions that reflect the participant’s experiences that they saw prescribing support a part of their role [45]. Stratton and Elia [46] advised the implementation of national and local policies, irrespective of treatment type, should be the priority, reflecting participants’ views that prescribing support takes place alongside training and standards implementation. This approach has been seen in other areas where prescribing savings have resulted in substantive dietetic funding [47, 48].

Most participants talked about empowering the MDT to drive efficiency. Strategies to encourage efficiency include sharing skills and enhancing the skills of others through delegation and training [5]. Delegation requires supervision to maintain patient safety, which was not discussed by participants [49]. Health care delegation is usually within teams and to lower or unqualified staff and so supervision requirements are usually established [5, 50]. As participants were not employed by the care homes, it is possible that supervision was not considered their role.

Many participants wanted to generate capacity to use their unique skills for residents with complex needs. This meets future NHS plans for MDT integration to provide a varied skill mix to meet patient’s needs [50, 51]. However, approximately 80% of older people have long-term and complex conditions [52] and 35% of residents are at risk of malnutrition [1]. The term complex is vague and could result in many residents being referred for a dietetic review, whereas most participants wanted to reduce referrals by delegating to the MDT. The dietitian’s skills need to be defined to establish whether the demand for service meets with dietetic capacity, as community workforce funding is limited [53].

Communication barriers were reported due to the MDT’s poor understanding of the dietetic role or nutritional care for older people. Ambiguity with roles and responsibilities can hinder MDT collaboration, but can be overcome by developing relationships and agreeing shared goals [54]. Weller *et al*. [55] agrees that communication improves when professionals work in partnership and respect each other’s priorities. Participants shared positive experiences using communication to improve the MDT’s understanding. Improving the visibility and understanding of the dietetic role is shared by other dietitians who wanted to be identified as the expert and leaders in nutritional care [56].

### Limitations

This research was undertaken as part of an MSc project by an inexperienced researcher (V.R.). To promote rigour, an experienced researcher (C.M.L.) provided oversight for the study. She independently coded all transcripts in full and peer-reviewed data analyses. It is considered a strength that the researchers provide different perspectives about the topic, V.R. is a dietitian with experience and knowledge of the topic area, whilst C.M.L. is an academic researcher who was able to question assumptions during analyses and provide a more independent viewpoint.

IPA guidelines recommend each data-set analysis completes the first four steps before moving on to another. Due to the researcher’s time limitations which included working full time in clinical practice, this was not always possible. Data analysis of each interview transcript was completed to step 3 before subsequent interviews and their analysis.

Guidance on how to complete data saturation in the literature is limited, it is understood that achieving data saturation may not have been fully achieved due to time constraints when completing an MSc research project. Data richness may have improved with longer interviews or a larger sample size [15]. However, the findings demonstrate that shared patterns of experiences were found to generate themes, which retained individual participant meaning. This represents IPA’s double hermeneutic, and idiography requirements were achieved [16]. Widening the study and including the MDT and resident perspectives may have added value; however, this was beyond the resources for this study.

Findings were not intended to be generalisable [18], they provide an understanding of the shared experiences of dietitians’ working with care homes in England.

## Conclusions

The findings suggest that community dietitians have a varied and significant role in care homes and highlight the need for MDT collaboration so that all residents receive quality nutritional care. There was recognition that the MDT could support dietetic capacity, thus allowing people requiring complex input better access to a dietitian. Further research is needed to understand the dietetic demand and capacity needs by care homes on a national level and the support needs of the MDT. The exploration of the MDT and resident perspectives would also provide a holistic view of the dietitian’s role and management of malnutrition in care homes. The need for an agreement of a defined role for dietitians working with care homes by the BDA is supported by this study.
